# “It’s for us –newcomers, LGBTQ persons, and HIV-positive persons. You feel free to be”: a qualitative study exploring social support group participation among African and Caribbean lesbian, gay, bisexual and transgender newcomers and refugees in Toronto, Canada

**DOI:** 10.1186/s12914-016-0092-0

**Published:** 2016-07-02

**Authors:** Carmen H. Logie, Ashley Lacombe-Duncan, Nakia Lee-Foon, Shannon Ryan, Hope Ramsay

**Affiliations:** Factor-Inwentash Faculty of Social Work, University of Toronto, 246 Bloor Street West, Toronto, M5S 1V4 ON Canada; Women’s College Research Institute, Women’s College Hospital, 790 Bay Street, 7th Floor, Toronto, M5G 1N8 ON Canada; Dalla Lana School of Public Health, University of Toronto, 155 College Street, Toronto, M5T 3M7 ON Canada; Black Coalition for AIDS Prevention, 20 Victoria Street, 4th Floor, Toronto, M5C 2N8 ON Canada

**Keywords:** Social support, LGBT, Refugee, Newcomer, Social determinants of health, SDOH, Mental health, Sexual and gender minorities

## Abstract

**Background:**

Stigma and discrimination harm the wellbeing of lesbian, gay, bisexual and transgender (LGBT) people and contribute to migration from contexts of sexual persecution and criminalization. Yet LGBT newcomers and refugees often face marginalization and struggles meeting the social determinants of health (SDOH) following immigration to countries such as Canada. Social isolation is a key social determinant of health that may play a significant role in shaping health disparities among LGBT newcomers and refugees. Social support may moderate the effect of stressors on mental health, reduce social isolation, and build social networks. Scant research, however, has examined social support groups targeting LGBT newcomers and refugees. The purpose of this qualitative study was to explore experiences of social support group participation among LGBT African and Caribbean newcomers and refugees in an urban Canadian city.

**Methods:**

We conducted 3 focus groups with a venue-based sample of LGBT African and Caribbean newcomers and refugees (*n* = 29) who attended social support groups at an ethno-specific AIDS Service Organization. Focus groups followed a semi-structured interview guide and were analyzed using narrative thematic techniques.

**Results:**

Participant narratives highlighted immigration stressors, social isolation, mental health issues, and challenges meeting the SDOH. Findings reveal multi-level benefits of social support group participation at intrapersonal (self-acceptance, improved mental health), interpersonal (reduced isolation, friendships), community (reciprocity, reduced stigma and discrimination), and structural (housing, employment, immigration, health care) levels.

**Conclusions:**

Findings suggest that social support groups tailored for LGBT African and Caribbean newcomers and refugees can address social isolation, community resilience, and enhance resource access. Health care providers can provide support groups, culturally and LGBT competent health services, and resource access to promote LGBT newcomers and refugees’ health and wellbeing.

## Background

Pervasive sexual stigma and discrimination are chronic, cumulative stressors that have deleterious health impacts among lesbian, gay, bisexual and transgender (LGBT) people [1–5]. Meyer’s minority stress model describes the role of distal processes, such as enacted stigma, whereby people experience acts of violence and unequal treatment (e.g., harassment), and perceived stigma, including concerns of rejection and negative treatment by others because of actual or perceived LGBT identity, in contributing to health disparities among LGBT people [3, 5, 6]. LGBT people may migrate to a new country due to sexual stigma, often including imprisonment, abuse, and threat of execution [7].

Upon migration, marginalization of LGBT people may be exacerbated for those experiencing intersecting stigma associated with sexuality, race, gender, class, and immigration status [8]. This intersecting marginalization among LGBT newcomers and refugees contributes to significant challenges in realizing the social determinants of health (SDOH) including meeting basic needs, such as secure housing and employment, and emotional wellbeing, including social support. A SDOH framework posits that the conditions necessary for health are shaped by individuals’ immediate, social, and political environments [9]. Newcomers and refugees frequently experience difficulties navigating Canada’s complex immigration processes, adapting to cultural norms, [10, 11] and being denied access to healthcare [12]. LGBT newcomers and refugees face additional challenges related to social isolation due to intersecting oppression based on race/ethnicity and LGBT identity, contributing further to health and mental health disparities. Social support may play a significant role in moderating the effects of sexual stigma on health among LGBT people [3, 5, 6], yet little attention has been afforded to strategies that may build social support among LGBT newcomers and refugees in the Canadian context.

Some studies suggest LGBT newcomers and refugees may not anticipate experiencing marginalization based on LGBT identity in Canada, as Canada is viewed as having human rights and protections for LGBT people, particularly compared to countries of origin where LGBT people may face punitive laws against homosexuality [13, 14]. Murray’s qualitative study with 54 LGBT refugee claimants in Toronto, Ontario found LGBT refugees’ expectations of safety and freedom, versus the realities of immigrating to Canada, including experiencing stigma and discrimination based on their LGBT status and race/ethnicity, contributed to disappointment and stress [14]. Addressing LGBT newcomer and refugee expectations, facilitating access to the SDOH, and reducing social isolation are important factors in optimizing the health and wellbeing of LGBT newcomers and refugees.

### Social support and LGBT newcomers and refugees

Meyer’s minority stress model posits that social support may buffer the effect of sexual stigma on LGB people’s mental health [3]. Social support may also reduce isolation, enhance feelings of belonging, and reduce the effects of discrimination among newcomers and refugees [15]. Social support groups can foster universality—the understanding that others have similar experiences [16]. Support groups among LGBT people have been described as creating environments that foster mentorship and contribute to positive identity development [17].

Social support groups may be therefore be particularly meaningful for African and Caribbean (AC) LGBT newcomers and refugees who experience marginalization based on LGBT identity, in addition to intersecting factors such as race and immigration status. In one study, social support was particularly critical in normalizing the intersection between ethno-racial and LGBT identities and traumatic experiences [11]. Although not LGBT-specific, studies with newcomers to Canada highlight the potential for support groups to address social isolation. Stewart et al. [15] explored social support among Chinese and Somali newcomers to Canada. They found that post-migration, newcomers faced challenges establishing and maintaining social networks, and that peers who had immigrated were an important source of support [15]. Stewart et al.’s [18] qualitative study explored the benefits of a support group for Somalian and Sudanese refugees [18]. Participants reported that the group reduced isolation, provided a place to express frustrations about structural barriers to navigating new systems, and fostered a kinship network [18].

Among scant research conducted with LGBT newcomers and refugees conducted in the U.S., studies demonstrate the importance of social support. A needs assessment with LGBT newcomers and refugees in Arizona highlighted a lack of LGBT competent health services, housing insecurity, and legal concerns [19]. Kahn [20] qualitatively explored the development of social connections among gender non-conforming refugees from Islamic countries seeking asylum in the U.S. due to persecution. Her study found that many participants were alienated from family and co-ethnic communities, and that service providers often served as ‘transitional kinship proxies’ [20]. Further studies are necessary to understand how social support groups may address intersecting oppression and resultant health and well-being disparities among LGBT newcomers and refugees, particularly within a Canadian context.

### Study objective

Scant studies have examined experiences of support groups among AC LGBT newcomers and refugees in Canada who may face intersecting forms of stigma and marginalization. The study objective was to explore experiences and perceived benefits of social support group participation among LGBT African and Caribbean (AC) newcomers and refugees in an urban Canadian centre.

## Methods

### Study design and sample

This study was a community-based partnership with an ethno-specific AIDS service organization (ASO) serving AC populations in an urban Canadian centre. The ASO implements monthly peer support groups for AC newcomers and refugees facilitated by a staff member. This ASO is the only ethno-specific ASO serving the AC community in the urban centre, providing services to community members who experience increased vulnerability to HIV due to social and structural drivers such as lack of access to adequate housing, financial, and social support, and intersecting stigma and discrimination. The ASO also serves members of the AC community who are living with HIV, although program attendance is not limited by HIV status.

We used venue-based sampling to recruit LGBT newcomers and refugees participating in at least one support group held by this ASO. One (group A) was developed for AC LGBT women, and a second (group B) for AC LGBT newcomers and refugees. The support groups are open for new members. Both group A and group B provide a space for informal discussions as well as organized workshops held by organization staff or outside expert facilitators that aim to address the SDOH, such as employment, immigration, and housing from a newcomer and refugee perspective, and LGBT health and human rights such as adoption for same sex couples. Both groups run monthly for a period of three hours. Participants were recruited for the study by the lead investigator (CHL) during the support groups, through the ASO’s email listserv, and word-of-mouth. Participants were eligible if they had attended group A and/or B at least once. A total of 29 participants participated in one of three focus groups (FG#1: *n* = 8, FG#2: *n* = 13, FG#3: *n* = 10) facilitated by trained doctoral students (ALD, NLF). Focus groups lasted 60 to 90 min and were conducted at the ASO. We held focus group sessions at the ASO to provide participants with a comfortable, easily accessible space where service providers were available in the event participants had questions or concerns about the topics discussed.

Prior to the focus group, we collected socio-demographic data including age, gender, sexual orientation, country-of-origin, immigrant/refugee status, and social support group attended using a brief form. We used a semi-structured focus group interview guide with open-ended questions to explore lived experiences of being a LGBT AC newcomer and/or refugee. We probed for participants’ experiences in the support groups, for example exploring motivation for involvement, perceived benefits, and recommendations. Sample questions include: “How did you hear about [group name]? What made you decide to attend the support group? What makes you keep coming to the support group? What have you found helpful about attending [group name]?” Focus group facilitators elicited feedback from all group members to ensure that all voices were represented. Participants received a $20.00 (CAD) honorarium for time and travel.

### Data analysis

Focus groups were digitally recorded and transcribed verbatim. Three investigators (CHL, ALD, NLF) independently analyzed transcripts using narrative thematic analysis to explore, analyze, and report themes. Narrative thematic analysis involves developing both inductive and deductive themes [21, 22]. Thematic analysis was conducted in a multi-step process that included multiple readings of the transcripts, team meetings to discuss initial findings, developing first level codes, developing themes by highlighting connections between codes, creating a thematic map, and finally refining themes and situating them in prior literature. In particular, we drew on a social ecological approach to develop overarching categories under which particular benefits to social support group participation are situated. Social ecological approaches recognize complex associations between social (e.g., friendships), structural (e.g., immigration) and intrapersonal (e.g., mental health) factors [23]. Trustworthiness of findings was established through debriefing with ASO managers and presenting findings at an ASO staff meeting for feedback [24].

## Results

Focus group participant socio-demographics are presented in Table 1. Participants were a mean age of 30.5 years [SD 8.0]. Most participants (89.6 %) identified as cisgender (non-transgender) men or women, with few participants identifying as transgender women. Participants identified as bisexual (42.9 %), gay (32.1 %), and lesbian (17.9 %). Notably, all participants from both group A, not specific to newcomers and refugees and group B, specific to newcomers and refugees, were born outside of Canada, in either Africa (29.6 %) or the Caribbean (70.4 %), and most came to Canada as refugees (79.3 %). Participants attended group B (64.3 %), group A (10.7 %) and both groups (25.0 %). Our analysis identified multi-level benefits of social support group participation at intrapersonal (*self-acceptance, improved mental health*), interpersonal (*reduced isolation, friendships),* community (*reciprocity, reduced stigma and discrimination*), and structural (*housing, employment, immigration, health care*) levels.

**Table 1:** Focus Group Participant Socio-demographic Characteristics

Characteristic	Mean (SD) or Frequency* (%)
Age (*n* = 26)	30.5 years (8.0)
Born in Canada (No)	100 %
Country of Birth (*n* = 27)	
African region	8 (29.6 %)
Caribbean region	19 (70.4 %)
Immigration Status	
Permanent resident	2 (6.9 %)
Visitor visa	1 (3.4 %)
Student visa	1 (3.4 %)
Refugee	23 (79.3 %)
Overstay (non-status)	2 (6.9 %)
Highest Level of Education (*n* = 28)	
Less than high school	1 (3.6 %)
Completed high school	6 (21.4 %)
Some college	7 (25.0 %)
Some university	3 (10.7 %)
Completed university degree (Bachelors)	6 (21.4 %)
Completed graduate degree	5 (17.9 %)
Employment Status (*n* = 28)	
Employed full-time	2 (7.1 %)
Employed part-time	2 (7.1 %)
Not employed: looking for work	5 (17.9 %)
Not employed: a student	2 (7.1 %)
Social assistance (ODSP, OW)	14 (50.0 %)
Unemployed	3 (10.7 %)
Annual Income (*n* = 16)	$13, 493 ($6984)
Sexual Orientation (*n* = 28)	
Heterosexual	1 (3.6 %)
Bisexual	12 (42.9 %)
Lesbian	5 (17.9 %)
Gay	9 (32.1 %)
Other	1 (3.6 %)
Gender Identity	
Cisgender male	15 (51.7 %)
Cisgender female	11 (37.9 %)
Transgender	3 (10.3 %)
Support Group(s) Attended (*n* = 28)	
Rainbow Sistahs	3 (10.7 %)
Foreign Integration	18 (64.3 %)
Both support groups	7 (25.0 %)

*All variables are for *n* = 29 unless otherwise noted due to missing data

### Intrapersonal benefits

#### Self-acceptance

Participants experienced increased acceptance of their LGBT identity and their rights as an LGBT person in Canadian society over the course of participating in groups. Self-acceptance was connected to participants’ experiences of feeling “welcomed”, “a part of something”, and being “treated like a human being”. A participant articulated the emotional process of being able to be open about their sexuality:

“I was shocked and really emotional. You are gay. You are lesbian. And you feel free to say that. It was my first, first, first experience here in Canada. And I think Canada is a free country because, back home, in Africa, it’s a big problem. You can never even open your mouth and say ‘I’m a lesbian’. Your family is going to kill you. The government is going to put you into prison”.

Another participant discussed meeting LGBT parents for the first time at a workshop held at the support groups, and how this expanded their understanding of possible family structures:

“Coming from a culture where gays don’t have certain rights, I never considered having a family or even going through the process of, let’s say, adopting. When I actually attended that workshop, you had a gay couple. It actually changed my mind about how I look at parenting.”

These narratives note the intersectional forms of discrimination (e.g., sexual orientation, class and gender) participants encountered in their own countries and how immigration to Canada not only enhanced their self-acceptance but ability to view themselves as having equal rights and opportunities as heterosexuals.

#### Improved mental health

Many participants described how their mental health improved after they began attending the support groups. One narrative reflected increased hope: “I love [the ASO]. The day I came, it was everything that was dead in me, it revived again. I started singing.” Others discussed attending groups more frequently to help them cope with challenging times: “there were times in my life, in those rough times, where I was frequent in those groups. I was really coming from a really bad situation”. Another participant discussed the benefits of having a community place that was always open to come to when feeling depressed:

“The community room that is open every day, all day, that is so innovative and useful… There were days I was really depressed and really lonely and can’t do anything. And you’re in bed all day. And you get out, you come here.”

### Interpersonal benefits

#### Reduced isolation

Many participants explicitly discussed that participating in the support groups reduced their isolation: “when you come to meetings, you see that you’re not really alone. You hear different people’s different stories and you can relate to it”. Others discussed feeling a sense of belonging and kinship in Canada after attending the support groups:

“It’s sort of finding a family. When I came here, it was winter time. Everybody was in their own house. It was like, oh, there is no life. But the day I met [group A], it was like chatting, laughing. It made me feel welcome in Canada.”

#### Friendships

Friendships developed through support group participation extended beyond the ASO to churches and the community-at-large. A participant noted: “I’ve met new friends here. I’ve strengthened relationships, as well, because I’m an LGBT refugee claimant. Many of us, we go to the same church.” Another narrative reflects the strength of the connections developed in the support groups:

“When you get out from [organization] and you walk to the subway and ride in a train with two or three people who have been to the session, that is when you hear what is going on in their lives and you get an understanding of what they are facing. A lot of times, you are able to help. I have ridden the train to the opposite direction of where I need to go, beyond where I need to get off, just talking.”

### Community

#### Reciprocity

Reciprocity, sharing knowledge and skills with others, stretched beyond the groups to the broader newcomer and refugee communities. For example, participants discussed sharing their transit-related knowledge with others—even strangers. In particular, many newcomers did not understand the public transportation transfer system:

“That is a thing that confuses a lot of people a lot of times. So, you need to explain to them and show them how to use the transfer because, sometimes, they get in conflict with the driver because the driver may not explain it. They just speak abruptly and the person feels mistreated. There are people that are not knowing the directions of the city. I’ve shown people how to get around the city and how to read the map. That takes some time. So, leaving this session and realizing what a person’s needs are, you jump in and you help them. People have done that to me and helped me. I do the same.”

This narrative reflects helping people in the larger community as a way of giving back. Moreover, participants indicated that group involvement motivated them to volunteer at the ASO and share their knowledge:

“Simple things like you want to find out how to do a work permit or how to get something off the computer or how to fax something or how to email something, you can ask one of these volunteers. You can come and ask us.”

#### Reduced stigma

Participants explained that the groups provided a safe space without fear of judgement based on sexual orientation, gender identity or HIV serostatus:

“It’s for us, as newcomers, to settle in because coming from our different countries, it accommodates LGBTQ persons as well as HIV [positive] persons. And you feel free to be among females, your peers. You don’t really have anybody being scornful of you if you are HIV. They interact with you just like you are a normal person and make you feel welcome. So, that is a good environment that you want to participate in or be a part of.”

Having a space free from stigma and discrimination was particularly important for people who never had a chance to learn about LGBT issues in their country of origin:

“Where I’m coming from there is no formal setting to educate a person within the LGBTQ community. With [group B], it is more of a formal setting. So, in that case, the information that you’re getting is from persons who actually live openly without feeling discriminated against. And so, you feel a little bit more accepted as to how it is that you, as an LGBTQ person, can actually integrate into this new lifestyle that you get on coming to Canada.”

These narratives highlight the groups as a forum where participants can learn from others how to navigate and overcome past experiences of stigma and discrimination based sexual orientation and/or gender identity. Learning how to navigate sexuality and gender identity with others sharing similar experiences contributed to participants feeling more accepted in Canadian society.

### Structural

Participants described how the groups increased their knowledge of, and access to, a range of resources and opportunities spanning from housing to health:

“Being new to Canada, you would want to come to a gathering to get information so that you can navigate through your new life, being a part of Canadian culture. A lot of things I have learned while coming to [group B], whether it be for housing, navigating the immigration process, health-related things as it relates to LGBTQ. And those things are things that affect us on a whole.”

This knowledge was an incentive for participation: “Each time, I keep coming, it’s like I’m getting knowledge and I’m learning more stuff. This makes me come back, back, back, back, back.”

#### Housing

Participants’ reported acquiring knowledge of housing rights: “There are some trouble landlords. You may not know your rights. This is what I’m supposed to do if my landlord acts this way. So, they made us understand our rights.” Another participant discussed a session on housing at one of the support groups that contributed to attaining stable housing: “I was in a shelter. I got a place.… I got that from the session about the housing.”

#### Employment

Participants discussed how the support groups offered volunteer opportunities and employment workshops that helped them attain employment in Canada. One of the participants volunteered at the ASO described: “Through my volunteering here, I was able to get job references for the job that I have now. And even through their employment workshop, that also helped me pick up the job I have right now.” Participants were also offered opportunities to participate in an HIV training certification; this could be included on the their resume and used as a skillset for future employment applications: “they did a workshop with us. And because of the content, the facilitators said that we can actually get certificates for it, which we did, and which I used to get me the job that I have right now. So, it was really helpful in that way.

#### Immigration

Participants reported receiving both emotional support as well as informational support to facilitate navigating the immigration process. A participant narrative articulated how emotional support helped reduce the stress of refugee claimants: “Those that have gone through [a refugee hearing] are usually a great encouragement to those who have not gone through it yet. And I can say, those who have not gone through are usually extremely stressed.” The support groups also provided information about the immigration process and connected participants with professionals such as immigration lawyers who could answer immigration-related questions: “they talk about different services, that we can acquire different services, that we can get into … they provide therapy, very good help, with ensuring that you have all the right information you need for your immigration process.”

#### Healthcare

Participants discussed the complexity of navigating the healthcare system as a newcomer to Canada. Participants noted the difficulties understanding what health care costs are covered in a universal healthcare system and how to gain access to physician services, medication and dentists. The groups helped clarify some of the questions around this complexity, enhancing participants’ ability to access healthcare:

“Whether you are a refugee claimant or a protected person, health is important. Maybe, come in and speak to persons who are new to Canada to say exactly how it is the benefits work in some cases, there are co-payments, there are coinsurance. There are little terminologies that we probably don’t understand or are probably different from where we’re coming from. So, in terms of health on a whole, and how it works here in Canada, I think that’s important, not just on STI and STDs, but health on a whole and how it is that health insurance helps to alleviate some of the costs.”

Others discussed being able to learn about LGBT sexual health issues at the groups: “We talk about sexual health, relationships between female and female, trans”.

## Discussion

African and Caribbean LGBT newcomers and refugees in an urban Canadian setting experience numerous stressors, including challenges navigating immigration and refugee processes, social isolation, and difficulties acquiring employment. Our findings suggest that social support groups tailored for LGBT AC newcomers and refugees can address social exclusion and help people meet the SDOH. Utilizing a combination of peer support and psycho-education, support groups tailored for AC LGBT newcomers and refugees can facilitate peer support and knowledge sharing that helps to build friendships, challenge stigma, and provide strategies for negotiating legal, employment, housing and health issues. Group participation revealed structural and social level benefits can contribute to improved intrapersonal factors, such as self-acceptance and mental health. While the ASO does not directly offer healthcare services, several participants discussed how the support groups provide individuals with the information needed to effectively navigate and access healthcare services. The groups’ provision of safe spaces enabled participants to discuss and learn about sensitive topics such as sexual health and healthy LGBT relationships under the guidance of trained service providers. Support groups also provide a space where individuals learn about their tenant rights and gain certifiable skills, thereby enhancing their SDOH (e.g., housing and employment).

The study ASO is the among the only organizations in the city that provide culturally relevant HIV prevention services made specifically for, and by AC populations. This specificity is necessary as the ASO support groups not only address the social contexts that contribute to disproportionate HIV infection rates among AC people in Canada—including racism—but also the challenges that AC LGBT newcomer/refugees may face navigating the refugee processes and sexual stigma. Its location is easily accessible by public transit and there were no age restrictions for attending the ASO support groups for ACB LGBT newcomers and refugees. These factors enhance accessibility for individuals of a wide age rage and from various parts of the city to attend; this also enabled our focus groups to include diverse participants and perspectives.

**Fig. 1:**
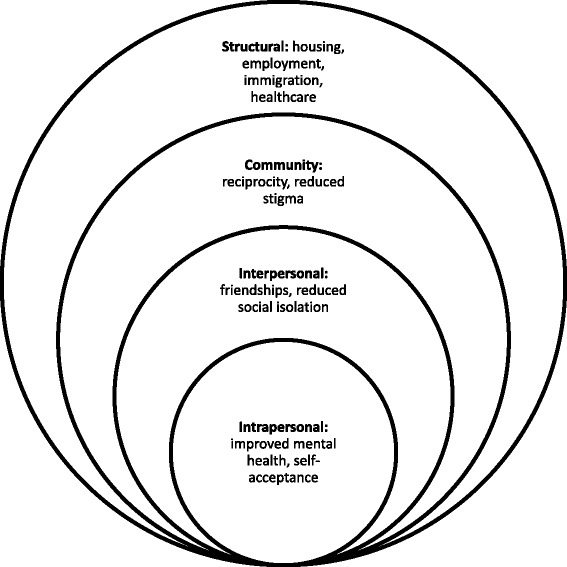
Conceptual model of a social ecological approach to understanding social support group benefits for African and Caribbean LGBT newcomers and refugees

A conceptual framework that incorporates our analytic themes is illustrated in Fig. 1. We categorized the themes and sub-themes within the social ecological model, reflecting intrapersonal (*self-acceptance, improved mental health*), interpersonal (*friendships, reduced social isolation*), community (*reciprocity, reduced stigma*) and structural (*housing, employment, immigration, health care*) domains. This model illustrates complex associations between social (e.g., friendships), structural (e.g., immigration) and intrapersonal (e.g., mental health) factors [23]. Findings highlight the utility of applying both social ecological analyses as well as Meyer’s minority stress model to understand benefits of social support groups for AC newcomers and refugees. Findings suggest social support groups can positively influence mental health as proposed by Meyer’s minority stress model. The processes by which social support groups benefit mental health and wellbeing are complex, mapping onto the social ecological model’s multiple levels. Social support appears to influence intrapersonal wellbeing by promoting self-acceptance that reduces internalized stigma; interpersonal benefits such as reduced isolation and friendships contribute to wellbeing. Community building and reducing perceived stigma through providing a safe space also improves mental health [3]. Our findings illustrate multifaceted benefits of social support groups that extend beyond intra/interpersonal domains to enhanced resource access. This model may be useful for future research and interventions focused on improving the SDOH among LGBT newcomers and refugees.

The current findings corroborate prior research that support groups with AC newcomers and refugees in Canada can reduce isolation, provide information, and build social networks [15, 18]. Similar to prior studies, participants described finding “family” and “universality” at the support groups [18, 20]. Our findings reflect research that consistently demonstrates the need to address SDOH for newcomers and refugees, including employment-oriented support [18–20]. Findings are also supported by work that highlights LGBT support groups can positively impact self-acceptance [17]. Most research on AC newcomers and refugee support groups has therefore focused on social and structural dimensions, while LGBT support groups have largely focused on intra/interpersonal dimensions. We build on this literature by demonstrating that support groups tailored for AC LGBT newcomers and refugees can address the intersection of needs and priorities associated with being a newcomer/refugee as well as needs of AC LGBT people. For example, AC LGBT newcomers and refugees face unique structural barriers related to newcomer/refugee status (e.g., barriers to accessing housing and healthcare) as well as sexual stigma (e.g., challenges with self-acceptance).

### Strengths and limitations

Limitations of the study include the focus group (FG) design, possibility of response bias, and sample. First, some participants may not have been comfortable speaking and sharing about their personal experiences in the FG setting [25]. Second, while FG co-facilitators were not associated with the ethno-specific ASO, participants may have responded more positively regarding the support groups due to the FG being located at the agency, and having other group participants present. To reduce response bias, the co-facilitators probed for divergent opinions and reinforced that responses were confidential. Finally, FG participants were connected to an ethno-specific ASO, which may have biased our sample in two ways. First, we may have under sampled LGBT immigrants and refugees who are most marginalized, evidenced by lack of attachment to an ASO. Although the ASO is the only service for AC people, some immigrants and refugees may have been averse to seeking services from an ethno-specific ASO due to HIV-related stigma. Further, some individuals may avoid the ASO for fear of having their sexual orientation and/or sexual identity ‘outed’ by accessing ASO services or by having contact with other ASO clients. Alternately, we may have oversampled AC LGBT newcomers and refugees experiencing life and immigration challenges such that they sought out support from a service agency. It is possible LGBT immigrants and refugees not seeking support from the ASO may have their social support needs met within their family, community and/or another organization. However, the validity of our findings is supported by the congruency between this study’s findings and prior research with LGBT people, newcomers and refugees. Moreover, given our small sample size we were unable to assess differences in experience with or benefits of social support groups for individuals based on differences in sexual and/or gender identities. Future studies should seek to understand the distinct benefits among diverse identities.

Despite these limitations, to our knowledge this is the first study to explore the benefits of social support group participation among AC LGBT newcomers and refugees. We develop a comprehensive understanding of the salience of support groups to the wellbeing of LGBT newcomers and refugees. Applying the social ecological model provides insight into the interactions between dimensions: support groups can have ripple effects that span from internal processes to resource acquisition. This study also provides researchers with a template which they can use to apply the minority stress model and social ecological approaches with diverse ethno-racial populations to better understand how social support groups can influence individuals, the SDOH, mental and overall health as well as their social and structural environments. Future research could use quantitative, longitudinal designs to explore the relationships between frequency and duration of support group participation and social, health and structural outcomes.

## Conclusions

Previous explorations of support groups among LGBT people, and newcomers and refugees, have fruitfully documented the positive impacts on health promotion, social networks, and acquisition of information [15, 17, 18]. Scant attention has examined support groups tailored for LGBT newcomers and refugees. This study expands on this literature to suggest that support groups may be particularly important for AC LGBT newcomers and refugees who often have limited family support, little exposure to other LGBT people, and experience intersecting forms of marginalization. The friendships developed in the groups can also act as alternate source of support and information that extend beyond the ASO monthly support group meetings. The conceptualization of *reciprocity*—wanting to give back support and knowledge to others—suggests support groups may play a role in building community resilience [26]. There may be a cyclical relationship between acquiring support that helps oneself adapt to challenges, and wanting to provide support to others experiencing similar adversities.

Data from this study and others highlight an urgent need for interventions to provide social support, and improve health services access, among LGBT newcomers and refugees [19, 20]. Social work and other advocates can provide emotional and information support during the immigration and refugee processes, and advocate for more just and humane practices [27]—and health care for refugee claimants. Health practitioners can support AC LGBT newcomers and refugees through providing access to support groups, culturally and LGBT competent mental health services, information and resources to realize justice and meet the SDOH.

## Abbreviations

AC, African and Caribbean; LGBT, lesbian, gay, bisexual, transgender; SDOH, social determinants of health
